# Application of Zinc-Based Metal-Organic Framework ZIF-8 on Paper: A Pilot Study on Visual Appearance and Effectiveness

**DOI:** 10.3390/polym17101369

**Published:** 2025-05-16

**Authors:** Eleonora Balliana, Mathilde Marchand, Valentina Di Matteo, Barbara Ballarin, Maria Cristina Cassani, Silvia Panzavolta, Elisabetta Zendri

**Affiliations:** 1 Department of Environmental Sciences and Statistics, Ca’ Foscari University Venice, Via Torino 155/b, 30170 Venice, Italy; mathilde22marchand@gmail.com; 2 Department of Industrial Chemistry “Toso Montanari”, University of Bologna, Via Piero Gobetti 85, 40129 Bologna, Italy; valentina.dimatteo5@unibo.it (V.D.M.); barbara.ballarin@unibo.it (B.B.); maria.cassani@unibo.it (M.C.C.); 3 Department of Chemistry “G. Ciamician”, University of Bologna, Via Selmi 2, 40126 Bologna, Italy; silvia.panzavolta@unibo.it

**Keywords:** paper application, metal–organic framework, ZIF-8, morphological characterisation, FTIR, XRF, XRD

## Abstract

Paper and cellulose-based materials are known for their sensitivity to humidity, which can create stresses among fibres and increase fragility. More importantly, humidity can lead to the formation of mould and stains, compromising both aesthetic value and long-term preservation, particularly for historical documents and books. This study explored the application of in situ prepared Zeolitic Imidazolate Framework (ZIF-8), a zinc-based MOF, on paper as a potential antimicrobial material. Hand-made and commercially printed papers were tested to assess the effective deposition and formation of the ZIF-8 network, with a focus on both visual appearance and physicochemical characteristics. X-ray fluorescence and diffraction, infrared spectroscopy, and scanning electron microscopy analysis confirmed the successful formation of the ZIF-8 network in all papers. The Zn content varied, as expected, depending on application time and paper characteristics. All treated papers exhibited minor variations in brilliance and showed slightly increased rigidity. The formation of white spots linked to Zn accumulation was observed, particularly in printed books where colourimetric and microscopic variations were more pronounced.

## 1. Introduction

Cultural heritage includes materials that have been used for centuries to convey both tangible and intangible aspects of our history and identity. This heritage encompasses historic buildings, paintings, textiles, and statues, as well as intangible heritage such as music, folklore, oral tradition, and traditional knowledge. Preserving these elements is a global societal and economic priority that must be shared and safeguarded by future generations. The deterioration of cultural heritage can result in substantial cultural and economic losses worldwide.

Alongside climate change, the biodeterioration of cultural heritage objects remains a persistent and unresolved challenge in conserving both organic and inorganic heritage materials [1,2]. Numerous solutions have been explored within the field of heritage conservation, but a definitive answer remains elusive. This challenge arises primarily from the need to balance the effectiveness of the proposed conservation methodologies with respect for and preservation of the original heritage materials [3,4,5,6]. While climate control and monitoring strategies can help mitigate biogenic damage to cultural assets, there is still a need to acquire resistance to antibacterial agents due to potentially inadequate storage conditions, dampness, and flooding [2,3,6,7,8].

Bacteria, fungi and spores are always present in the environment, and they can proliferate rapidly, leading to significant damage when optimal growth conditions are met. Therefore, it is essential to explore more suitable and flexible solutions that focus on preventing long-term biofilm growth while considering ecotoxicology and practical applications in heritage preservation [2,5,8]. Several factors (i.e., constituent materials, conservation history, storage environment) significantly influence the choice of appropriate indirect solutions and/or the selection of an ideal biocide for direct intervention in preserving artworks.

Various solutions have been explored for the preservation of artworks. These include commercially available biocides tailored for the heritage field [9,10,11], treatments involving specialised radiation and alcohols [12,13], nanotechnologies [6,14,15,16,17], and the more recent applications of essential oils [10,18,19]. Notably, over the past decades, nanomaterials and modified polymers have been extensively proposed as antimicrobial agents in water disinfection, food packaging, and healthcare applications [2,20,21,22]. Recently, metal–organic frameworks (MOFs) have gained attention for their applications not only in biomedicine and food packaging [2,23,24,25,26,27,28], but also in the heritage field [2,29,30,31,32,33]. Specifically, Zn-based nanoparticles, along with the more recent Ag- and Cu-based MOF composites, have demonstrated promising results for antibacterial applications [14,34,35,36,37,38,39].

Books and cellulose-based artworks are particularly vulnerable to bacteria and mould growth among the heritage materials. Paper, documents, and books—even those that are newly printed—are susceptible to fluctuations in humidity, which can lead to microbial proliferation. This can result in irreversible damage and staining, ultimately causing the loss of precious and invaluable artefacts [3,40,41,42]. Furthermore, the growth of microorganisms can contribute to the acidification of paper and increased fragility, further compromising the future preservation of these artworks [40,43,44].

Products incorporating nanoparticles and MOFs have been tested for paper conservation [30,32,45]; however, clear and practical strategies are still lacking. This is partly due to the need for compatibility, aesthetic safety, appropriate application methodologies, and long-term effectiveness. Additionally, heritage materials, such as books, are often unique, exhibiting distinct characteristics, including specific chemical and physical properties, conservation history, and manufacturing techniques [5,8,46]. These factors, along with environmental influences that significantly affect the presence and growth of microorganisms, complicate the successful use of even the most promising products for paper conservation.

Current research in antimicrobial products for preventative paper conservation has shown great interest in multifunctional metallic nanomaterials. Among the materials tested, Ag-based composites, Zn oxides (ZnOx), and MOFs have been successfully evaluated for paper conservation, particularly in removing acid and Volatile Organic Compounds (VOCs) [29,32,33].

ZnO is one of the most common nanomaterials used as an antimicrobial product, exhibiting greater safety and long-term stability than organic antibiotics. ZnO has also been applied in modern papermaking products to provide biological resistance. In the same way, and more importantly, MOFs have attracted significant attention in antimicrobial activities in biomedical, industrial fields, and air purification [36,47,48].

MOFs represent a unique class of ordered nanoporous solid crystals that have garnered significant attention due to their large surface area, low toxicity and high porosity [47,48,49]. MOFs are constructed from inorganic metal ions/clusters and organic ligands, obtaining multifunctional materials due to their unique advantages of large pore volume, great specific surface area, abundant active sites, and hydrophilic/hydrophobic behaviours. MOFs have been demonstrated, moreover, to possess superb performance in terms of structure and property flexibility, easily obtaining composite according to specific requirements [50]. These aspects show MOFs’ extraordinary potential for heritage preservation.

Among MOFs, zeolite imidazolate frameworks (ZIFs) have gained interest in heritage applications. ZIFs have a topological crystal structure like those found in aluminosilicate zeolites [4]. In a ZIF composite, the zeolite framework, composed of tetrahedral silicon or aluminium atoms, is typically bridged by oxygen and connected by transition metals such as Zn or Co, along with imidazole (Im) as linkers. The hybrid framework of ZIFs offers greater flexibility in surface modification, often allowing rational tuning of surface properties compared to zeolites, along with better thermal, hydrothermal and chemical properties than other MOFs [51]. An example of a MOF is Zeolitic Imidazolate Framework-8 (ZIF-8), which consists of 2-methylimidazole and zinc ions. ZIF-8 is known for its chemical stability in aqueous and basic environments and has a surface area of approximately 1800 m^2^/g [50,51,52]. The crystal structures of ZIFs share topological similarities with aluminosilicate zeolites [51,53]. ZIF-8-based composites exhibit intrinsic antibacterial properties, effectively inhibiting the proliferation of external microorganisms and have been successfully applied in the medical and food-packaging fields [52]. Zinc ions released by ZIF-8 at pH levels below 6.5 exhibit excellent antibacterial activity, primarily due to its highly porous structure functioning as a reservoir for Zn metal ions. Zn ions are known to act as a potential antimicrobial agent as they can induce cell deformation and rupture of the bacteria cell membrane, resulting in bacterial death. Zn ions can also interfere with protein synthesis, inhibiting bacterial division and proliferation. Zn ions can, moreover, increase the generation of reactive oxygen species or create an alkaline microenvironment, leading to the inhibition of bacterial growth or death [36].

These favourable physical properties, along with excellent thermal stability and pH-responsive dissolution behaviour in acidic solutions, have prompted investigations of ZIF-8 in various fields, including gas capture and storage, catalysis, sensing, biomedical applications [24,28,50,53,54], and cultural heritage [29,30]. ZIF composites can be engineered to control and calibrate their ion release, creating new and flexible applications in the heritage field as well, where antibacterial and antifungal preventive solutions are often demanded.

This study examines the in situ formation of ZIF-8 on various paper substrates, focusing on morphological and visual aspects, as well as chemical and structural characteristics. To gain a comprehensive understanding of the formation and interaction of ZIF-8 on paper, a combination of techniques was employed, including microscopic observations, colourimetric analysis, attenuated total reflection infrared spectroscopy (FTIR-ATR), X-ray fluorescence, X-ray diffraction (XRD), and scanning electron microscopy (SEM).

## 2. Materials and Methods

### 2.1. Sample Selection and Description

In this study, six types of paper were selected to explore potential variations in ZIF-8 network formation in relation to application times and paper characteristics. Industrial and hand-made papers often differ in morphology, appearance, and porosity; these factors might affect product efficiency and use. For this purpose, old book paper (both printed and unprinted) and new handmade paper were employed to assess the suitability of the product for in situ paper applications.

The hand-made papers were prepared by a paper conservator following traditional techniques; they are characterised by small inhomogeneities and a rougher surface when compared to commercially available materials. The papers were produced using high-quality fibres employed in paper conservation and purchased by Gangolf Ulbricht Papier (Berlin, Germany); no additives or fillers were added. The hand-made papers have a similar thickness with a grammage ranging between 170 and 200 gsm (grams per square metre); the bleached hemp was slightly different, with a value of around 250 gsm. The commercial paper book had, as expected, a grammage of about 70 gsm, typical of regular printing paper. The industrial paper was taken from a book printed around the late 1960s, selecting printed and non-printed pages. The chemical nature of the thin glossy coating characterising the commercial paper was explored by Infrared Spectroscopy (see Supplementary Materials). A total of 60 samples (4 × 4 cm) were prepared, with 10 replicates for each material (3 per application time) and identified as reported in Table 1; 9 were treated and one was kept as a reference (NT—untreated). The samples were investigated before and after the ZIF-8 application to check the distribution of the products and possible physical-chemical modifications.

### 2.2. In Situ Synthesis of ZIF-8 and Application on Paper

The deposition of ZIF-8 on paper supports was carried out at room temperature, using in all experiments ultrapure water purified with the Milli-Q plus system (Millipore Co, resistivity over 18 MΩ cm, Burlington, VT, USA). Chemicals employed in the synthesis were used as received from Sigma Aldrich (now Merck KGaA, Darmstadt, Germany).

A stoichiometric Zn/2H-mIM (2-methylimidazole) ratio of 1:10 was chosen to reduce the possible alteration of the support. Immersion times of 2, 4, and 6 h were tested to verify product distribution and possible material variations. All paper samples were weighed before and after the product application to verify the weight increase due to the deposition of the coordination compound. After weighing, the paper samples were placed in weighing boats and 10 mL of a 0.273 M solution of Zn(OAc)_2_∙2H_2_O (2.73 mmol), prepared by dissolving 600 mg of salt in 10 mL of water, was added. After waiting for about 30 min (the time necessary for the zinc ions to be absorbed into the support), 10 mL of a 2.70 M solution of 2H-mIM (27.0 mmol), prepared by dissolving 2.24 g of the organic linker in 10 mL of water, was added to carry out the synthesis in situ of ZIF-8. The syntheses were carried out in triplicate inside weighing shuttles as the reaction solution covered the sample completely. After the application, the samples were thoroughly washed with water to neutralise the basic pH; approximately 6–7 washes per sample were performed. Finally, the excess of 2H-mIM still present within the ZIF-8’s pores was eliminated by sublimation, keeping the samples at 100 °C and 10^−2^ mbar. Once completely dry, the samples were weighed again to verify the possible weight variation due to the coordination compound’s deposition. The possible effect of just the solvent 2-methylimidazole for the in situ formation of ZIF-8 was tested on each material in three replicates to assess potential paper degradation. The supernatant suspensions, remaining in the weighing shuttle, were washed with water and centrifuged (Centrifuge Remi XSR-8D, 6000 rpm for 20 min, REMI Elektrotechnik LTD, Mumbai; India). The collected ZIF-8 powders were dried at 60 °C for 24 h and, after being thermally activated at 100 °C and 10^−2^ mbar, were stored in a desiccator and subjected to PXRD analysis to assess the composite’s crystalline phase.

For comparison, a sample of ZIF-8 was prepared as follows: 10 mL of an aqueous solution of Zn(OAc)_2_·2H_2_O (2.73 mmol) was added to a solution of 2-methylimidazole (10 mL, 27.30 mmol). The resulting suspension (Zn^2+^:2-HmIM = 1:10) was stirred (400 rpm) at room temperature for 6 h. Then, it was centrifuged and the solid was washed with water until the washings had a pH of ca. 7 (litmus paper) before air drying in an oven at 70 °C for 24 h. After thermal activation at 100 °C and 10^−2^ mbar, the white powder was stored in a desiccator.

Table 2 reports the weight variation expressed as percentage variation (%W) using the formula: (W_f_ − W_i_)/W_f_)) × 100; W_f_ represents the final weight of samples after treatments at different application times, and W_i_ is the initial weight of the samples. Papers (indicated as R_name_2h, 4h, 6h) were subjected to the in situ formation using only the solvent (2-methylimidazole) for the different application times, showing minimal weight variation. Most of the samples show an increase in weight, which is maximum at 4 h, while at 2 h, the variation is, for most papers, challenging to appreciate. Surprisingly, the longer application time (6 h) yielded values that were often lower or comparable to those of the 4 h, possibly indicating the papers’ saturation.

### 2.3. Analytical Techniques and Papers Characterisation

As previously said, all papers were subjected to a multi-analytical approach before and after the MOF application to check possible variations, particularly in terms of morphology and aspects. The final aspect of an object is crucial in the heritage field, and it is often considered superior to the actual efficiency of a product [8,46,55].

Microscopic observations of the samples were performed with a portable microscope manufactured by Dino-Lite Digital Microscope (Almere, The Netherlands), equipped with visible and UV-light sources and a camera. A mask was used to take 8 measures on each sample: 4 with natural light and 4 with UV light (390–400 nm emission), at two different magnifications (55× and 225×). Possible morphological variations due to the ZIF-8 treatments were discussed compared to the images taken before and after three application times: 2 hours (2 h), 4 hours (4 h), and 6 hours (6 h). Pictures were elaborated with DinoCapture 2.0 software. Additional microscopic observations of the samples were also realised with a ZEISS SteREO Discovery.V8 optical microscope (ZEISS, Berlin, Germany) equipped with a ZEISS Axiocam 208 colour at different magnifications (1×, 3.2×, 8×). The observations were mainly performed to highlight possible morphological variations such as product accumulations, degradation of paper fibres, etc.

A KONICA MINOLTA CM-2600 d/2500 spectrophotometer (KONICA MINOLTA, Rotterdam; The Netherlands) equipped with CM-S100w SpectraMagicTM NX 6 software was employed to evaluate chromatic variation. A D65 standard [56] illuminate, 8-degree viewing angle geometry and a 3 mm diameter target area were used. The colourimetric data are discussed here in terms of application time. Measurements were performed in the CIELAB1976 space, and data were collected in SCI and SCE modalities (specular component included and excluded, respectively) since both can provide information about morphological changes (smoother/rougher surface) and molecular chemical changes that can promote colour variation [57]. The total colour variation, expressed as ΔE, was calculated by Formula (1):

(1) $$∆E=\sqrt{{∆L}^{*2}+{∆a}^{*2}+{∆b}^{*2}}$$

where ΔL*, Δa* and Δb* are the differences between the average calculated for the same sample before and after the ZIF-8 application.

The resulting SCI/SCE values were the average of five independent points, and the related standard deviation was also calculated. ∆L*, ∆a*, ∆b*, and ∆E* were calculated to the coordinates before the application. According to literature, when the colour difference (ΔE*) is less than three, the changes are not detectable by the human eye. A ΔE* value greater than three signifies a visible colour difference that is still deemed acceptable. However, when ΔE* exceeds five, the change in colour is considered unacceptable for artworks [58].

FTIR measurements on papers before and after ZIF-8 application were realised with an ALPHA II spectrometer by Bruker Optics (BRUKER, Ettlingen, Germany), equipped with an ATR modulus with a single-bounce diamond. Spectra were recorded from 4000 to 400 cm^−1^ with a resolution of 4 cm^−1^, 64 scans per acquisition. Five independent points were collected per sample, and one single analysis was performed on the ink specifically for the printed book. The background was acquired before each analysis and automatically subtracted by the software OPUS 9.0. All spectra were first analysed using proprietary software and further elaborated with Origin 9.5 regarding peak position and absorbance. All data were normalised to 1 and compared to the non-treated samples (NT) and pure synthesised ZIF-8 spectra for peak identifications.

A CRONO Bruker XRF spectrometer (BRUKER, Munich, Germany) assessed the ZIF-8 distribution on papers after applications based on Zn distribution. At least five independent points were considered on the treated paper. Point analysis was recorded with a 15 kW Voltage, 100 μA current, 0.5 mm collimator size and an acquisition time of 40 s. Data were first evaluated using ESPRIT proprietary software (version 2.2.1.4280) and then elaborated with Origin 9.5 regarding repeatability, accuracy, and possible differences according to application time. Next to Zn, Ca, K, Si, Mg and Fe were considered as elements commonly present in paper materials [59].

Powder X-Ray Diffraction (PXRD) patterns were recorded in reflection mode using a Philips X’Celerator diffractometer (Malvern Panalytical, Milan, Italy) equipped with a graphite monochromator to check ZIF-8 correct formation after in situ synthesis. The 2θ range was from 4 to 45° with a step size of 0.100° and a time per step of 10 s. CuKα (40 mA, 40 kV, 1.54 Å) was applied. PXRD patterns were recorded directly on the papers (before and after treatments) and on the supernatant dry residue in the weighing shuttle (see Section 2.2). Data were preliminary treated with proprietary software X’ Pert Data Viewer version 2.2 and elaborated with Origin 8.5; pure ZIF-8 PXRD spectrum was run for peak identification.

Morphological investigations were also performed using a Scanning Electron Microscope (SEM), and the images were obtained using a Leica/Cambridge Stereoscan 360 (Oxford Instruments, Abingdon, UK) (vacuum 2 × 10^−5^ Torr; filament voltage 300 Volts; filament current intensity: 2.7 A) with INCA software; INCA Digimizer software (version 5.8.0, MedCalc Software Ltd., Ostend, Belgium) was used to estimate the mean dimensions, averaging the measurements over at least 100 data points per sample. Paper samples were coated with Au before analysis to reduce material charge EDX analyses were performed on uncoated samples by means of an ESEM Zeiss EVO LS 10 (ZEISS, Munich, Germany) equipped with EDS Bruker Quantax System (BRUKER, Munich, Germany).

## 3. Results and Discussion

### 3.1. Morphological Aspect of Papers After ZIF-8 Application

**Figure 1:**
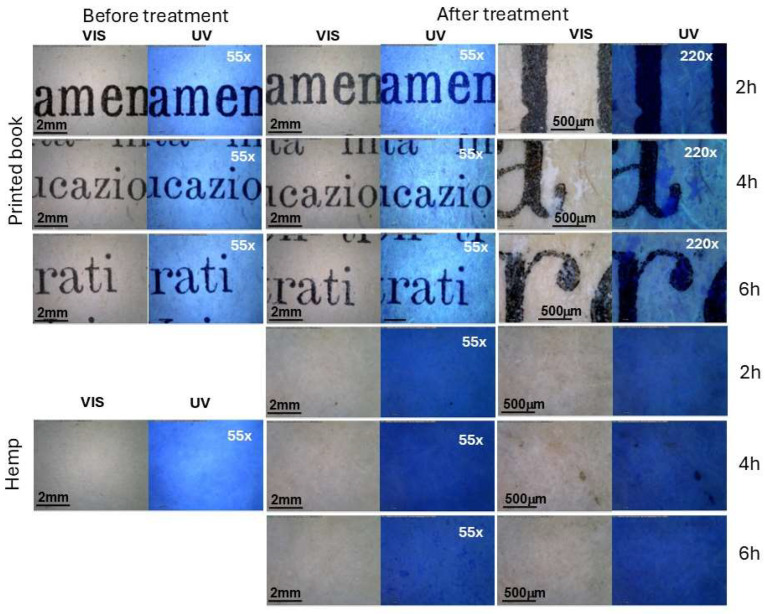
Paper samples obtained for the printed book and hemp observed via contact microscope (50× and 220×) before and after treatment with ZIF-8 solution 2 h, 4 h, and 6 h under visible (VIS) and ultraviolet light (UV).

Morphological differences were visible between non-treated and treated samples (Figure 1). In general, the application of ZIF-8 changed the visual appearance of the sample, regardless of the type of paper and application times; all samples appeared less bright at both magnifications (55× and 225×) after the ZIF-8 application. The hand-made papers showed small white spots, more evident under UV in the case of the brown ones (hemp and mixt linen-hemp), possibly due to zinc accumulation or deposition. The white spots were, however, clearly evident on the book pages, increasing the ZIF-8 application time (Figure 1). In the printed book, Zn tends to form well-defined spots that, in some cases, partially cover the ink. The evident deposition of Zn in the book papers, with respect to the hand-made paper, was probably facilitated by the paper’s coating. The commercial print book papers are brown and characterised by a thin gloss coating, likely resulting from the mixing of additives with a binder, such as starch, according to the literature (see Supplementary Materials). Applying a coating is typical in commercial paper and is used to increase the optical and printing properties of the paper in industrial serial printing [60]. Minor fading and ink detachment were also observed in a few points on the printed book (Figure 1), possibly due to the water contact rather than the treatments themselves. Hand-made papers are more porous, and the ZIF-8 might be absorbed more homogenously, as also evidenced under UV at higher magnification, at least at 2 h, with the formation of smaller white spots at 6 h application (Figure 1). Few images obtained with the contact microscope for the other papers are reported in Figures S1 and S2 (in Supplementary Materials).

The samples in contact just with the 2-methylimidazole solvent solution (indicated as R_name_2h, 4h, 6h) did not show significant variation at least when observed at 55× and 220× either under visible and UV light (Figures S3 and S4 in Supplementary Materials). In the specific case of the printed books, the basic 2-methylimidazole water solution (pH 12) do not seem to damage the ink even after 6 h application times.

**Figure 2:**
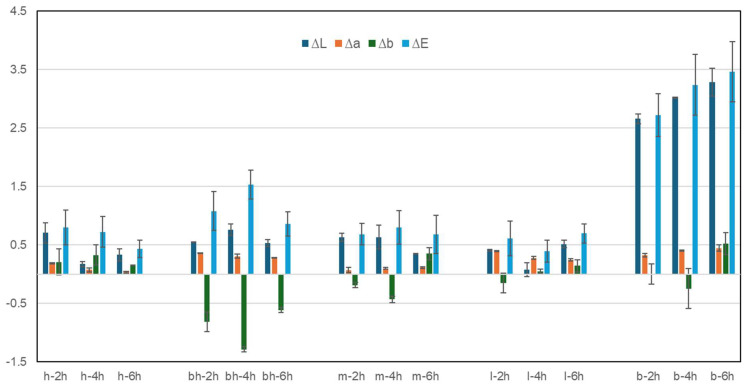
Variations in the chromatic coordinates L*, a*, b* and ΔE of all paper samples (h—hemp; bh—bleached hemp; m—mix hemp/linen; l—linen; b—book) after treatments at 2 h, 4 h, 6 h. Data are the average values of 10 points on three replicates per type and application time.

Colourimetric measurements were taken before and after ZIF-8 application to better explore aesthetic variations on both sides of the papers. ΔE* and colourimetric coordinated (L*a*b*) were considered and calculated. In this case, the printed book samples were excluded as measuring a spot only related to the ink or the paper was impossible due to the instrument measurement area. The papers subjected to the contact with the solvent also exhibited minimal colour variations, which resulted in more evidence for the book but were still undetectable by the human eye (Figure S5 in Supplementary Materials). It is possible to note a variation in the coordinate b* that tends to negative values (blue shit) for the bleached hemp, the mixed hemp/linen and linen paper. Figure 2 reports the values calculated for the papers after 2 h, 4 h, and 6 h treatments. In general, all the hand-made papers do not show significative colour variation with ΔE* below 3, indicating a change that is not detectable by human eyes.

Regardless of the application time, as expected, the book presented the greatest chromatic variations with an increase in L* (whitening) and ΔE*, with values around 3, indicating visible variations that became more evident according to the application time. The ΔE* variations were 2.72 for 2 h, 3.24 for 4 h and 3.47 for 6 h application time. Values around 3 in ΔE* are still considered acceptable in the heritage field as limits of human detectability. Noteworthy is the fact that the reference samples subjected solely to the blank solutions exhibit ΔE variations of approximately 3, with increasing values directly related to the application time. This may indicate that the variation is mainly associated with a washing effect caused by the water-based solution. Nevertheless, as previously said, in the case of the paper book, white spots must be considered a side effect. The paper surface coating on the book pages probably favoured the accumulation of Zn on the surface. Therefore, strategies to improve ZIF-8 distribution should be formulated, at least in the case of coated papers. According to Table S1 (in Supplementary Materials), the values of ∆L* show an increase in brightness (>L*) on almost every sample and minor variation for ∆a* and ∆b* with a higher sensitivity for the b* coordinates. Specifically, the bleached hemp paper (bh) showed a consistent variation for the b* coordinates towards negative values (−*b > blue) among the hand-made papers; this was also observed for the mixed paper realised combining linen and bleached hemp.

### 3.2. Evaluation of ZIF-8 Presence After Application via FTIR-ATR and XRF

The FTIR-ATR spectra of pure synthesised ZIF-8 and papers are resumed in Figure S6, while Figure S7 (in Supplementary Materials) reports the papers after the contact with the solvent 2-methylimidazole evidencing no residues or chemical modification.

**Figure 3:**
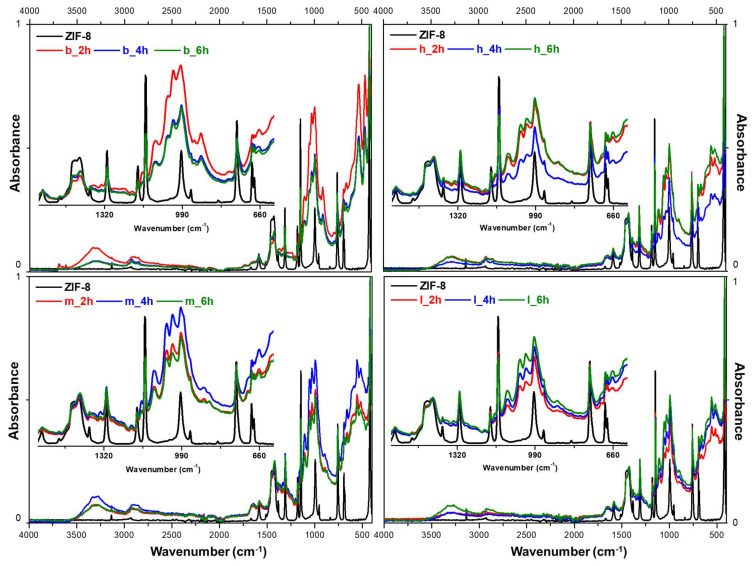
FTIR-ATR spectra obtained for the printed book and three types of hand-made paper (hemp, linen and a mixture of the two) after applying ZIF-8 at 2, 4 and 6 h. A detail is given for each group between 1600 and 600 cm^−1^. All spectra in absorbance mode were normalised to a scale of 0 to 1.

After application, paper samples were subjected to FTIR-ATR characterisation to evaluate the formation of ZIF-8 in relation to the type of paper and application time. Figure 3 shows the FTIR-ATR spectra collected for the printed book and three types of hand-made paper (hemp, linen and a mixture) against the pure spectrum of ZIF-8.

Despite the prominent signal given by the main absorption peaks of cellulose [61,62], all spectra evidenced the presence of ZIF-8 thanks to its typical vibration bands, which become clearly defined, increasing the application time. On the treated samples, already after the 2 h application time, it is possible to observe the presence of the imidazole ring (at about 1430 cm^−1^, 1146 cm^−1^, 990 cm^−1^) and, in a few cases, at an increasing intensity with the application time of the C=N bending at about 1540 cm^−1^. The additional characteristic band occurring due to the stretching of the Zn-N is also present, but not consistently—it is detectable in a few cases [24,63]. It is interesting to notice how the formation of ZIF-8 seems more homogeneous for the hand-made papers than the printed book confronting the ATR of multiple samples and points. In both cases, the 4 h application achieved the most homogeneous results, with a lower signal variability (Figure S8 in Supplementary Materials). Nevertheless, a longer application time might favour a deeper absorption of the products within the cellulose fibre.

**Figure 4:**
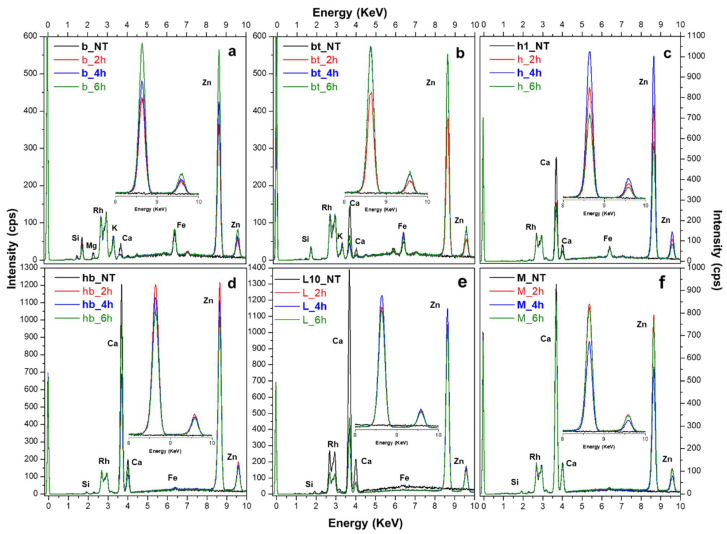
XRF spectra of all papers ((**a**) book, (**b**) printed book, (**c**) hemp, (**d**) bleached hemp, (**e**) linen, (**f**) mixed hemp/linen) after 2, 4 and 6 h application time versus the corresponding untreated material (NT).

X-ray fluorescence analyses were also performed on the treated paper to obtain a deeper understanding of the ZIF-8 distribution within the material; at least five points per sample were checked and averaged (Figure 4).

In comparison to the samples before treatment (NT), next to typical absorption peaks related to paper (Si, Ca, K and traces of Mg in a few cases), the collected XRF spectra (Figure 4) show the prominent XRF peaks of Zn at about 8.6 eV (Kα) and 6.9 eV (Kβ). In this case, as the contribution given by the paper is almost irrelevant, it is possible to better appreciate, at least in the case of the book, the correlation between the increase in Zn intensity and increasing application time (Figure 4a,b).

In the case of the hand-made papers, establishing a clear correlation between the amount of zinc (Zn) and the application time proves to be challenging. XRF and FTIR-ATR are, in fact, point analysis techniques and not quantitatively precise. Nevertheless, by considering at least 10 points per sample, the average values can provide valuable insights for evaluating the distribution of ZIF-8. For instance, hemp paper (Figure 4c) shows an inverse relationship, showing the lowest Zn intensity with the longer application time. Overall, a 4 h application time appeared to be the most effective, often yielding Zn content comparable to the 6 h application time.

**Figure 5:**
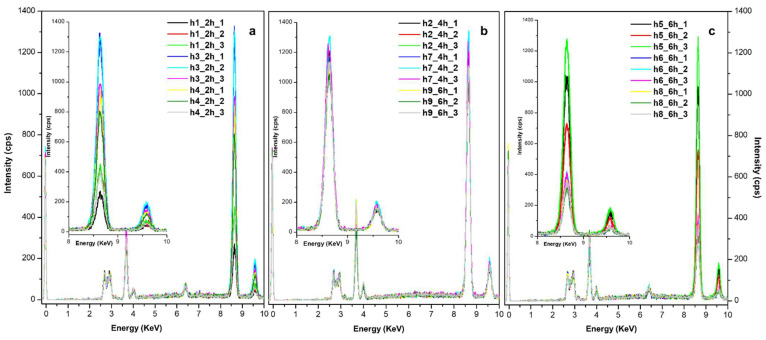
Evaluation of Zn variability on XRF spectra collected for the hemp samples in different spots at 2 h (**a**), 4 h (**b**), and 6 h (**c**) application time.

When different spots and samples are considered, there is, as expected, an inevitable variability in Zn intensity due to possible inhomogeneities in the product formation or the type of papers. Figure 5 presents, as an example, the XRF spectra obtained for the hemp samples.

At 2 h and 6 h applications (Figure 5a,c), there is a marked variation in Zn intensity among the replicates and the considered spots. This variation is drastically reduced at 4 h (Figure 5b), reaching a Zn intensity that is almost the same at 6 h. An application time of 4 h seems sufficient to achieve a fair content in Zn; reducing application time could be an advantage for avoiding colour variation and product accumulation.

### 3.3. Assessment of ZIF-8 via SEM and XRD Analysis

**Figure 6:**
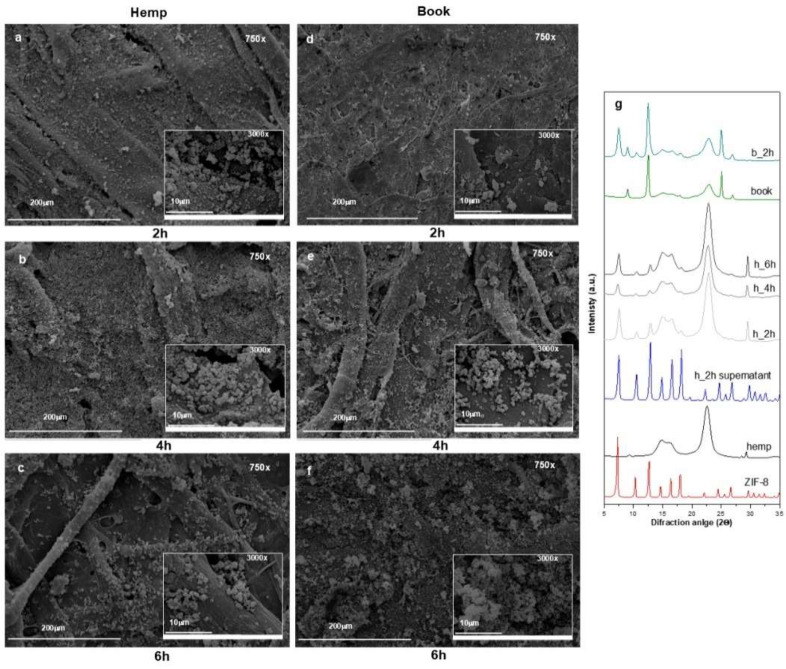
SEM images obtained for hemp and book after ZIF-8 in situ synthesis: (**a**) hemp after 2 h (750×, 3000×), (**b**) hemp after 4 h (750×, 3000×), (**c**) hemp after 6 h (750×, 3000×), (**d**) book after 2 h (750×, 3000×), (**e**) book after 4 h (750×, 3000×), (**f**) book after 6 h (750×, 3000×); (**g**) XRD patterns of ZIF-8, pure hemp and treated samples at different application times; ZIF-8 recovered from the supernatant after 2 h, untreated book and book after 2 h.

To better understand ZIF-8 distribution and crystal morphology, SEM and XRD analyses were performed on the papers treated at different application times. Figure 6 shows the results obtained for the hemp and paper book samples as representative examples.

Confronting the SEM images, it is possible to observe an increase in ZIF-8 crystals with application time. At 2 h application time, the deposited ZIF-8 presents an inhomogeneous size distribution and, particularly for the book, a poorly defined morphology (Figure 6d). On the contrary, at 4 h, both paper substrates appear homogeneously covered, showing the characteristic rhombic dodecahedron morphology of sodalite-ZIF-8 (Figure 6b,e), with a uniform size distribution and dimensional range of 0.5–0.9 μm, in line with what was found in our previous works [24,53].

On the contrary, after 6 h application time, it is possible to evidence an uneven distribution of ZIF-8 that tends to aggregate, creating accumulation spots (Figure 6c,f), probably responsible for the white spots visible on the papers. This behaviour is quite evident in the book (Figure 6f), where the morphology of the crystals also appears less defined. In the latter case, the paper coating may be partially responsible for this phenomenon, reducing the penetration of ZIF-8 within the paper fibres. Based on these observations, 4 h of contact time seems sufficient for an even and homogenous ZIF-8 distribution. As expected, the material characteristics (such as production processes, thickness, preparation) play an important role in the distribution and interaction of ZIF-8 within the cellulosic matrix.

To evaluate the fair distribution of ZIF-8 on the papers, selected areas were investigated using EDX, with a focus on the presence of Zn. SEM-EDX analysis of the book and hemp papers revealed a homogeneous formation of the MOF within the materials (Figure S9 in Supplementary Materials). EDX spectra assessed the presence of Zn, and its distribution across the samples, evaluated by collecting maps, appeared relatively homogeneous despite variations in deposition times. ZIF-8 is most likely absorbed within the materials rather than simply deposited on the surface.

The PXRD spectra performed directly on the composites were compared with the diffraction pattern of the powders recovered after centrifugation from the supernatants (as described in Section 2.2) and with a sample of ZIF-8 appositely prepared for comparison. In Figure 6g, from the bottom to the top, the following diffractograms are reported: pristine ZIF-8, untreated hemp, ZIF-8 recovered from the supernatant, hemp modified with ZIF-8 at all three application times, book paper, and book modified with ZIF-8 after 2 h. The supernatant shows all the main diffraction peaks of the pristine ZIF-8 at 2θ = 7.3°, 10.4°, 12.7°, 14.7°, 16.4°, and 18.0° associated with the crystal planes (110), (220), (211), (220), (310), and (222), confirming the formation of the sodalite phase [64,65].

The XRD spectra obtained for the treated papers showed only a few characteristic peaks of the sodalite phase (at about 2θ = 7.3°, 10.4°, 12.7°) as part of the XRD pattern was covered by the paper signal. Nevertheless, ZIF-8 is present, and the correct sodalite ZIF-8 crystal phase formed under the applied conditions.

The papers subjected to contact with the blank solution containing only 2-methylimidazole were also examined by SEM-EDX to check for possible material degradation at different magnifications (1500×, 3000×). The collected SEM images did not show any evidence of possible fibre degradation, even after the longer application time of 6 h (Figure S10 in Supplementary Materials).

## 4. Conclusions, Final Remarks, and Future Work

The conservation of heritage patrimony consistently requires efficient and sustainable solutions for the preservation and safekeeping of materials. Each heritage material often needs specific temperature, humidity, and air quality specifications for a proper conservation. As observed with antimicrobic materials, the methods and materials available frequently apply only to specific substances and have limited efficacy or functionality over time. Inorganic metal oxides, especially zinc oxide (ZnO), are effective for preserving cellulose-based materials like paper. ZnO acts as an antimicrobial agent, providing resistance to biological degradation. It exhibits antifungal activity against strains such as Aspergillus, Saccharomyces cerevisiae, and Rhizopus, which are often found in archives and museums.

Metal–organic frameworks (MOFs) have attracted significant attention, primarily in the biomedical field and later also in heritage applications for stone conservation, air purification, and paper conservation. MOFs display excellent performance with flexible structures and properties, existing in nanoforms that harness the benefits of size, surface, and quantum effects, along with unique advantages such as multiple functions and controllable properties not typically found in other nanomaterials. The antimicrobial applications of ZIF-8 and ZIF-8-based composites show significant potential, superior to traditional Zn-based nano or composite materials, and have been successfully used as implant coatings, water filtration, and multifunctional fabrics.

This work evaluates the potential application of in situ prepared Zeolitic Imidazolate Framework (ZIF-8), a zinc-based MOF, on paper. The primary focus was to understand the formation and compatibility of the products, assessing the morphological and visual characteristics of the papers after treatments, which are critical factors, often detrimental to heritage applications.

All papers exhibited minor colour variation after treatment, indicating possible use of ZIF-8 on paper. The ZIF-8 distribution appears related to the morphological characteristics of the papers. White spots were particularly noticeable in the printed books due to Zn accumulation. The coated book displayed the most significant issues, likely due to a very thin surface coating (most probably kaolin mixed with starch), which made the paper surface glossy and less permeable compared to the other papers. FTIR-ATR and XRF analyses demonstrated that ZIF-8 efficiently formed on paper in varying amounts based on the application time and paper characteristics. A 4 h application resulted in the best distribution of the product and crystal formation for both handmade and industrial paper, often yielding superior results compared to a 6 h application. At 4 h, at least for the tested papers, the formation and distribution of ZIF-8 is relatively homogeneous on and within the papers, reaching almost a saturation effect of the actual Zn that can be effectively formed or accepted by the paper. A longer application time could promote accumulation spots with undesirable aesthetic variations and affect the fibre’s morphological integrity and mechanical resistance. The creation of inhomogeneous product formation could also impact the antimicrobial action and the conservation of the paper itself over time.

The research showed that ZIF-8 can be successfully formed in situ on paper samples using a simple and easily reproducible protocol, suggesting a potential safe use of the materials. These results represent a preliminary step in exploring the potential application of the Zeolitic Imidazolate Framework (ZIF-8) for paper applications. Nevertheless, as evidenced by the printed book, some criticisms need to be addressed, particularly regarding the homogeneous distribution of the product on coated papers, which are often found in archives and collections. A thin coating (i.e., animal glue or starch), whether originally present or applied during conservation treatment, is quite common in historical papers to reduce porosity and enhance paper strength, etc.

The efficient formation and distribution of the products are essential factors, along with support after treatment in heritage applications. The possibility of eventually spraying the products or gently removing any excess before the paper samples are completely dry could help reduce visual inhomogeneities. Testing the ZIF-8 at other concentrations and on a wider variety of samples should also be considered to align the potential antimicrobial action with aesthetic necessity. Nevertheless, considering that paper-based materials (i.e., painted manuscripts, books, paper-based artistic materials) are so peculiar that instead of focusing mainly on paper characteristics (colour, presence of coating, conservation history, etc.), optimising the application protocol (i.e., application methods and ZIF-8 concentration) is the leading strategic choice to minimise issues related to paper characteristics. Reducing the influence of aspects such as paper porosity, roughness, or density, which may affect the distribution and formation of the products, could ensure safe use regardless of the paper characteristics and the possible extension of ZIF-8 and related MOFs to the industrial fields.

Once the protocol is checked, assessing the actual antimicrobial properties of the formed ZIF-8 is crucial, possibly selecting common biodeteriogens found in archives and collections. Equally important would be testing the efficacy over time and in unstable environmental conditions, such as historic buildings.

## Figures and Tables

**Table 1:** Sample description and identification IDs.

Sample ID	Description
Hemp—h	Pure hand-made hemp paper
Bleached Hemp—bh	Pure bleached hand-made hemp paper
Mix Hemp-Linen—m	Mixed hand-made hemp (60%) and linen (40%) paper
Linen—l	Pure hand-made paper linen
Book—b	Commercial-coated paper from the late 1900
Printed book—pb	Commercial-coated paper from the late 1900

**Table 2:** Weight variation on the paper samples at the different application times: W_f_—final weight of samples after treatments at different application times, W_i_—initial weight of samples; weight variation %W = (W_f_ − W_i_)/W_f_)) × 100.

	Application Time 2 h				Application Time 4 h				Application Time 6 h			
Type of Paper	Sample Id	W_i_	W_f_	%W	Sample Id	W_i_	W_f_	%W	Sample Id	W_i_	W_f_	%W
Book—b	R_b20_2h	0.116	0.115	−1	R_b23_4h	0.116	0.115	1	R_b26_6h	0.121	0.120	0
	R_b21_2h	0.117	0.116	−1	R_b24_4h	0.120	0.120	0	R_b27_6h	0.118	0.117	−1
	R_b22_2h	0.115	0.115	0	R_b25_4h	0.119	0.118	1	R_b28_6h	0.115	0.115	0
	b1_2h	0.149	0.149	0	b9_4h	0.146	0.154	5	b3_6h	0.142	0.146	3
	b7_2h	0.110	0.112	2	b4_4h	0.136	0.143	5	b2_6h	0.153	0.159	4
	b8_2h	0.109	0.110	1	b6_4h	0.117	0.124	6	b5_6h	0.095	0.100	5
Printed book—pb	R_pb20_2h	0.117	0.117	0	R_pb23_4h	0.103	0.102	1	R_pb26_4h	0.109	0.109	0
	R_pb21_2h	0.115	0.113	−1	R_pb24_4h	0.122	0.121	1	R_pb27_4h	0.103	0.103	0
	R_pb22_2h	0.120	0.120	0	R_pb25_4h	0.109	0.109	0	R_pb28_4h	0.107	0.106	−1
	pb5_2h	0.133	0.133	0	pb2_4h	0.175	0.181	3	pb3_6h	0.183	0.194	6
	pb6_2h	0.131	0.132	1	pb1_4h	0.131	0.135	3	pb9_6h	0.167	0.176	5
	pb4_2h	0.174	0.177	2	pb7_4h	0.132	0.140	6	pb8_6h	0.135	0.139	3
Linen—l	R_l20_2h	0.260	0.260	0	R_l23_4h	0.304	0.301	0	R_l26_6h	0.370	0.365	−1
	R_l21_2h	0.358	0.349	−2	R_l24_4h	0.348	0.346	1	R_l27_6h	0.351	0.350	0
	R_l22_2h	0.320	0.320	0	R_l25_4h	0.321	0.321	0	R_l28_6h	0.362	0.361	0
	l5_2h	0.305	0.309	1	l2_4h	0.306	0.319	4	l6_6h	0.309	0.310	0
	l3_2h	0.312	0.321	3	l1_4h	0.330	0.341	3	l7_6h	0.270	0.279	3
	l9_2h	0.380	0.381	0	l8_4h	0.318	0.328	3	l4_6h	0.409	0.419	2
Hemp—h	R_h20_2h	0.300	0.299		R23_h_4h	0.341	0.340	1	R26_h_6h	0.321	0.321	0
	R_h21_2h	0.334	0.329		R24_h_4h	0.282	0.279	0	R27_h_6h	0.344	0.342	0
	R_h22_2h	0.304	0.303		R25_h_4h	0.340	0.340	0	R28_h_6h	0.322	0.320	0
	h4_2h	0.237	0.237	0	h9_4h	0.247	0.254	3	h8_6h	0.235	0.236	0
	h3_2h	0.248	0.254	2	h2_4h	0.269	0.274	2	h5_6h	0.233	0.239	3
	h1_2h	0.269	0.271	1	h7_4h	0.245	0.256	4	h6_6h	0.250	0.250	0
Bleached Hemp—bh	R_bh20_2h	0.435	0.434	0	R_bh23_4h	0.451	0.448	1	R_bh26_6h	0.334	0.333	0
	R_bh21_2h	0.463	0.459	−1	R_bh24_4h	0.419	0.419	0	R_bh27_6h	0.403	0.401	0
	R_bh22_2h	0.443	0.443	0	R_bh25_4h	0.433	0.432	0	R_bh28_6h	0.402	0.404	0
	bh2_2h	0.409	0.417	2	bh9_4h	0.441	0.451	2	bh1_6h	0.368	0.373	1
	bh6_2h	0.586	0.591	1	bh4_4h	0.540	0.551	2	bh5_6h	0.504	0.513	2
	bh7_2h	0.475	0.476	0	bh8_4h	0.535	0.559	4	bh3_6h	0.441	0.448	2
Mixed hemp/linen—m	R_m20_2h	0.261	0.261	0	R_m23_4h	0.250	0.249	0	R_m26_6h	0.242	0.240	−1
	R_m21_2h	0.183	0.181	−1	R_m24_4h	0.275	0.274	0	R_m24_6h	0.281	0.280	−1
	R_m22_2h	0.231	0.231	0	R_m25_4h	0.265	0.264	0	R_m28_6h	0.247	0.247	0
	m2_2h	0.281	0.284	1	m3_4h	0.226	0.230	2	m4_6h	0.264	0.267	1
	m6_2h	0.201	0.201	0	m1_4h	0.256	0.261	2	m5_6h	0.274	0.275	0
	m8_2h	0.282	0.284	1	m7_4h	0.259	0.267	3	m9_6h	0.255	0.257	1

## Data Availability

The original contributions presented in the study are included in the article/Supplementary Materials; further inquiries can be directed to the corresponding authors.

## Supplementary Materials

The following supporting information can be downloaded at: https://www.mdpi.com/article/10.3390/polym17101369/s1. Figure S1: Paper samples obtained for the book and linen, observed via contact microscope (50× and 220×), before and after 2 h, 4 h, and 6 h treatment with ZIF-8 solution under visible (VIS) and ultraviolet (UV) light; Figure S2: Paper samples obtained for mixed hemp/linen and bleached hemp, observed via contact microscope (50× and 220×), before and after 2 h, 4 h, and 6 h treatment with ZIF-8 solution under visible (VIS) and ultraviolet (UV) light; Figure S3: Paper samples subjected to the blank experiment, observed via contact microscope (50× and 220×), before and after 2 h, 4 h, and 6 h after the blank experiment (10 mL solution of 2-HmIM 27.0 mmol) under visible (VIS) and ultraviolet (UV) light; Figure S4: Paper samples subjected to the blank experiment, observed via contact microscope (50× and 220×), before and after 2 h, 4 h, and 6 h after the blank experiment (10 mL solution of 2-HmIM 27.0 mmol) under visible (VIS) and ultraviolet (UV) light; Figure S5: Variations in the chromatic coordinates L*, a*, b* and ΔE of all paper samples (R_h—hemp; R_bh—bleached hemp; R_m—mix hemp/linen; R_l—linen; R_b—book) undergone the blank experiment (10 mL solution of 2-HmIM 27.0 mmol) after 2 h, 4 h, and 6 h contact. The data represent the average values of 10 points from three replicates per type and application time; Figure S6: FTIR-ATR spectra of all considered paper and ZIF-8 to identify the main characteristic peaks; Figure S7: FTIR-ATR spectra obtained for all papers subjected to the blank experiment (R_h—hemp; R_bh—bleached hemp; R_m—mix hemp/linen; R_l—linen; R_b—book) after 6 hours application time. 2-metyilimidazole and the hemp spectra are reported for comparison; Figure S8: Comparison of FTIR-ATR spectra obtained for multiple points and samples of the printed book and hemp after 2, 4, and 6 h of treatment with ZIF-8. All spectra in Absorbance mode were normalised to a scale of 0 to 1; Figure S9: SEM images, Zn distribution maps and EDX spectra on a selected area obtained for hemp and book after 2, 4 and 6 h application time; Figure S10: Confront of SEM images (1550×, 3000×) obtained for all papers before (NT) and after 6 h contact with the solvent (10 mL solution of 2-HmIM 27.0 mmol); Table S1: Variations in the chromatic coordinates L*, a*, b*, and ΔE of all paper samples after treatments. All samples were measured on the front and back, considering at least 10 points on three replicates per paper type and application time. The value collected for the papers subjected to the blank experiment are reported as R_name_2/4/6h. References [24,60,61,62,63,66] are cited in Supplementary Materials.
