# Supplementary material for: Application of Zinc-Based Metal-Organic Framework ZIF-8 on Paper: A Pilot Study on Visual Appearance and Effectiveness
Source: Polymers (Basel). 2025 May 16;17(10):1369. doi: 10.3390/polym17101369 (PMC12114989; doi:10.3390/polym17101369)
Supplement: Supplementary file 1 [file polymers-17-01369-s001.zip › polymers-3601591-supplementary.pdf]

Supplementary Material

Application of Zinc-based Metal-Organic Framework ZIF-8 on paper: A Pilot Study on Visual Appearance and Effectiveness

Eleonora Balliana [1*], Mathilde Marchand [1], Valentina Di Matteo [2], Barbara Ballarin [2], Maria Cristina Cassani [2], Silvia Panzavolta [3], Elisabetta Zendri [1*]

[1] Department of Environmental Sciences and Statistics, Ca' Foscari University Venice, Via Torino 155/b, 30170 Venice, Italy; eleonora.balliana@unive.it, mathilde22marchand@gmail.com , elizen@unive.it

[2] Department of Industrial Chemistry "Toso Montanari", University of Bologna, Università di Bologna, Via Piero Gobetti 85, I-40129 - Bologna, Italy; valentina.dimatteo5@unibo.it; barbara.ballarin@unibo.it; maria.cassani@unibo.it

[3] Department of Chemistry "G. Ciamician", University of Bologna, Via Selmi 2, I-40126 - Bologna, Italy; silvia.panzavolta@unibo.it

[*]Corresponding authors: eleonora.balliana@unive.it; elizen@unive.it

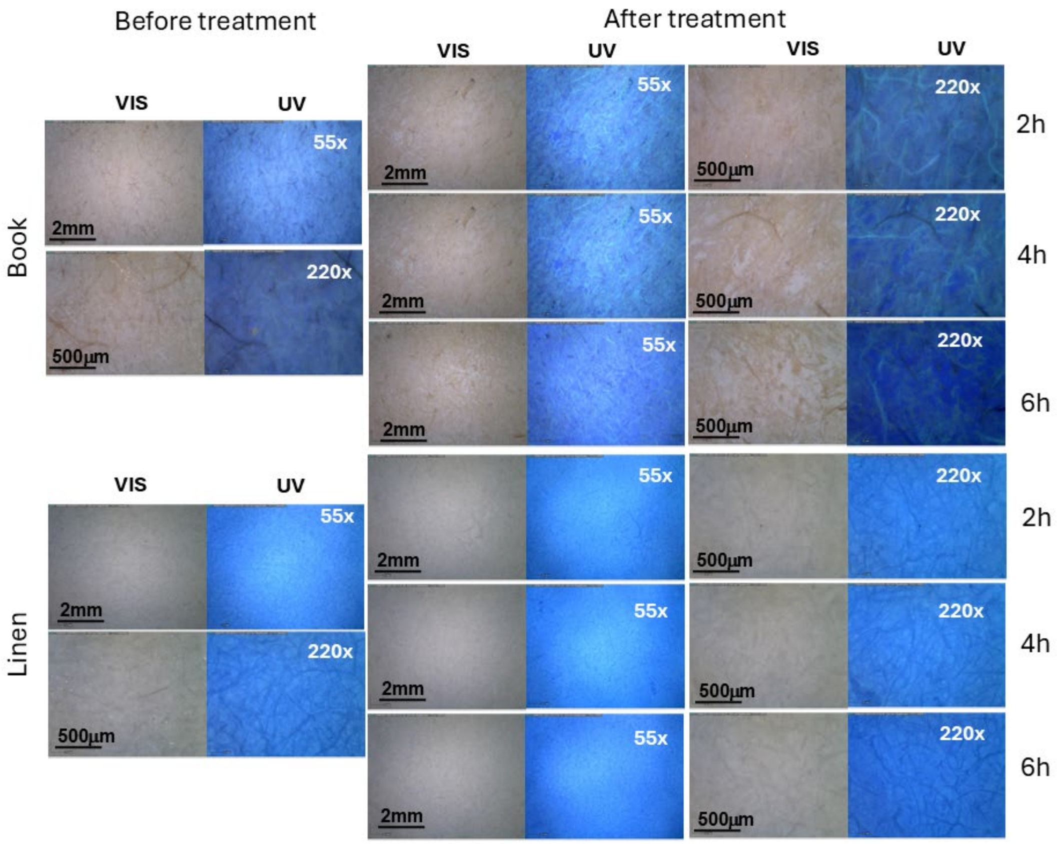

Figure S1. Paper samples obtained for the book and linen, observed via contact microscope (50x and 220x), before and after 2h, 4h, and 6h treatment with ZIF-8 solution under visible (VIS) and ultraviolet (UV) light.

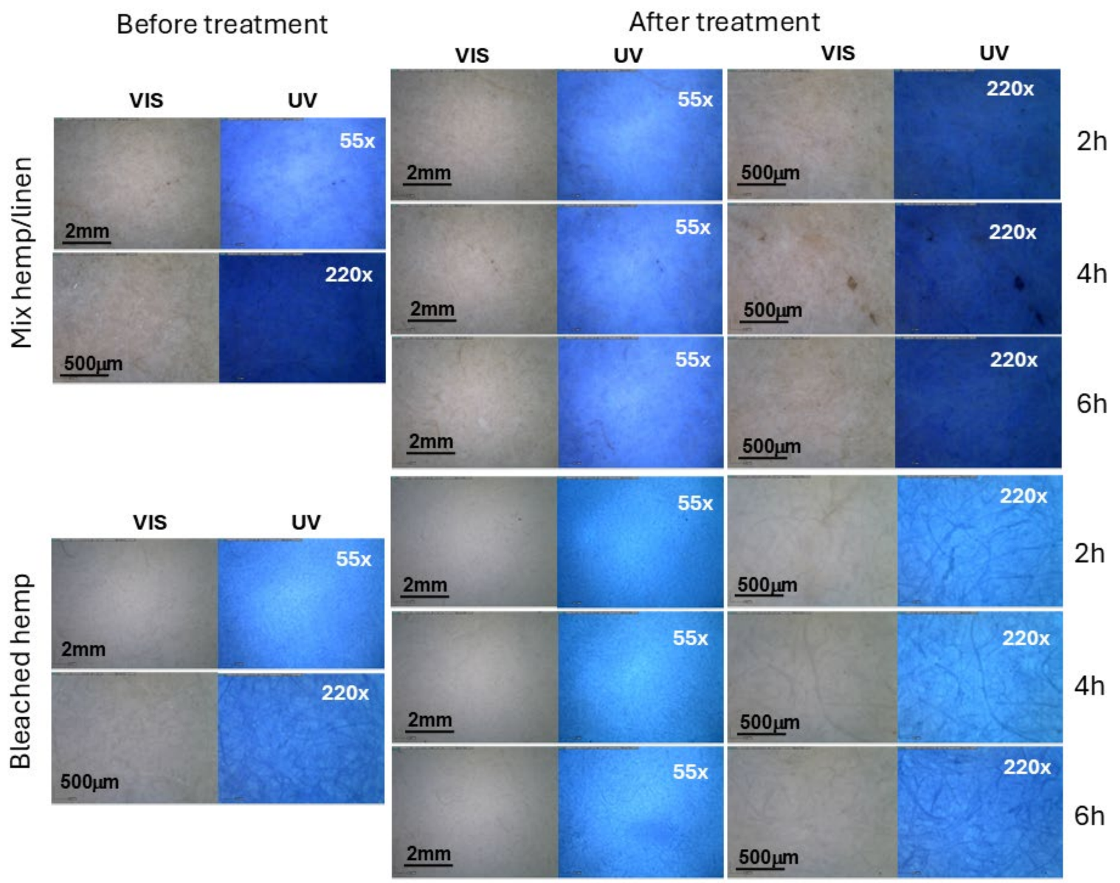

Figure S2. Paper samples obtained for mixed hemp/linen and bleached hemp, observed via contact microscope (50x and 220x), before and after 2h, 4h, and 6h treatment with ZIF-8 solution under visible (VIS) and ultraviolet (UV) light.

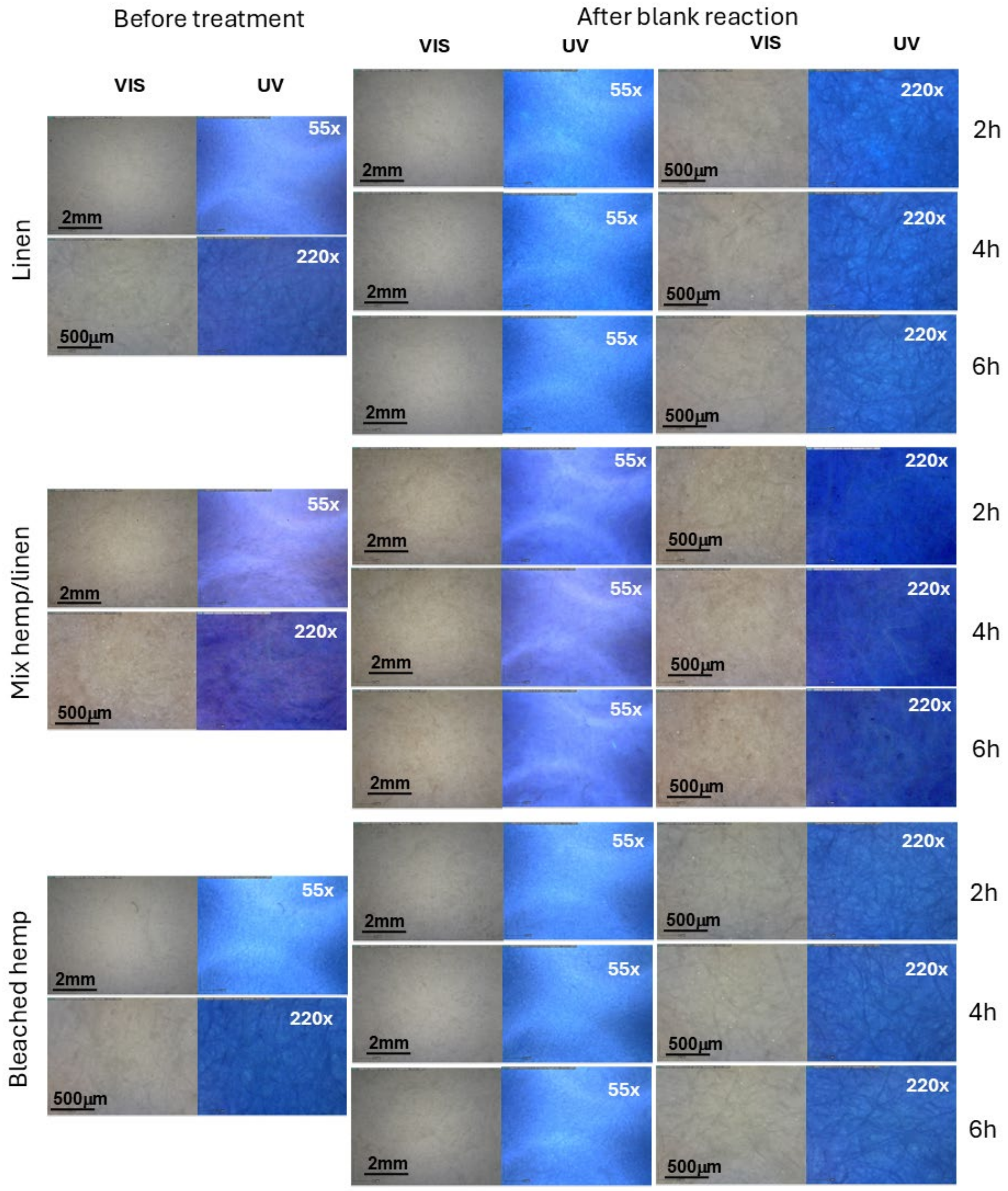

Figure S3. Paper samples subjected to the blank experiment, observed via contact microscope (50x and 220x), before and after 2h, 4h, and 6h after the blank experiment (10 ml solution of 2-HmIM 27.0 mmol) under visible (VIS) and ultraviolet (UV) light.

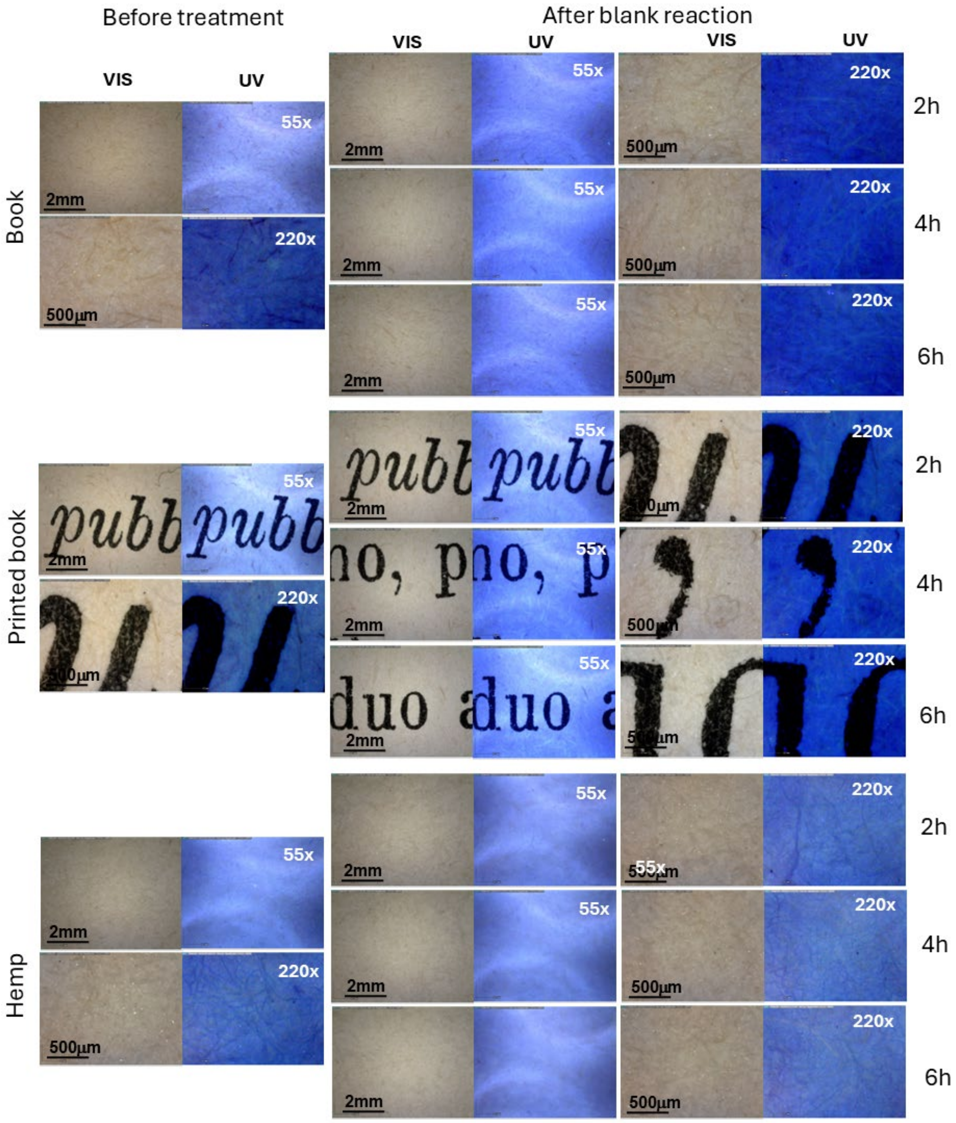

Figure S4. Paper samples subjected to the blank experiment, observed via contact microscope (50x and 220x), before and after 2h, 4h, and 6h after the blank experiment (10 ml solution of 2-HmIM 27.0 mmol) under visible (VIS) and ultraviolet (UV) light.

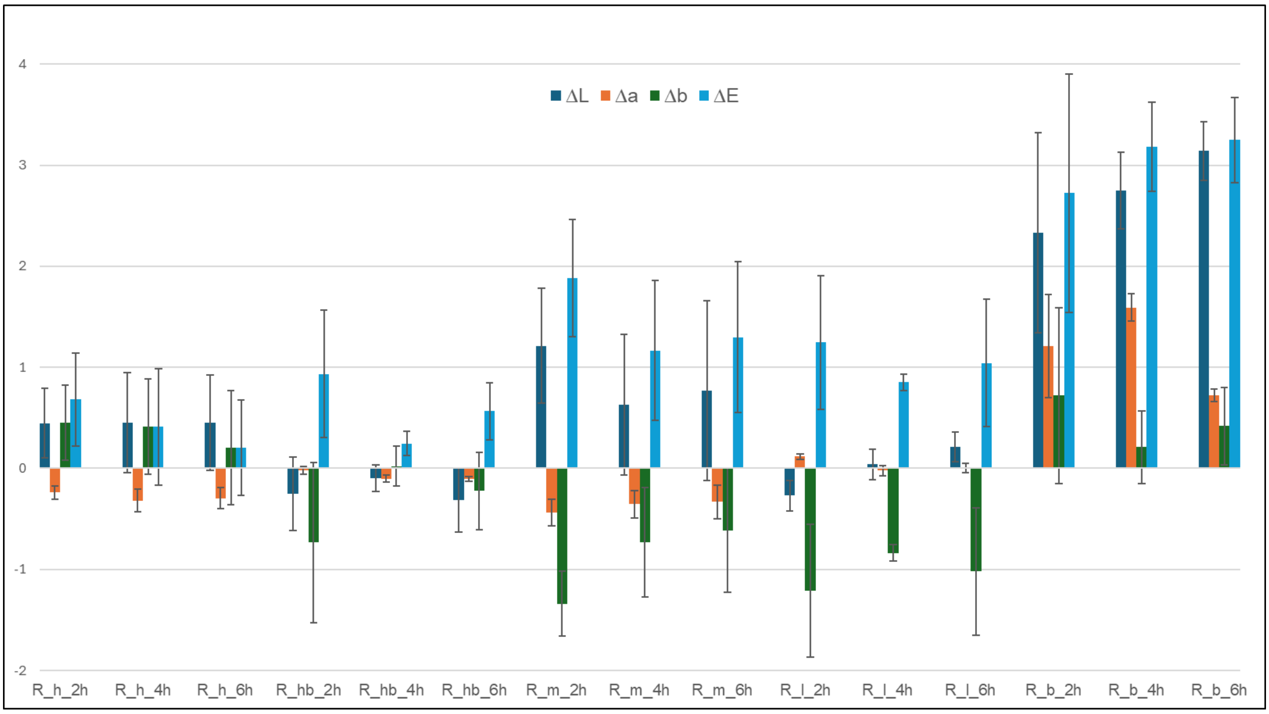

Figure S5. Variations in the chromatic coordinates L*, a*, b* and ΔE of all paper samples (R_h- hemp; R_bh- bleached hemp; R_m – mix hemp/linen; R_l – linen; R_b – book) undergone the blank experiment (10 ml solution of 2-HmIM 27.0 mmol) after 2h, 4h, and 6h contact. The data represent the average values of 10 points from three replicates per type and application time.

FTIR-ATR of untreated paper and pure ZIF-8

The chemical composition of the paper samples was assessed before and after ZIF-8 applications. Figure S1 reports the FTIR-ATR spectra of the different papers and the pure ZIF-8. All spectra exhibit similar absorption peaks, primarily related to cellulose and hemicellulose, with minor differences likely attributed to the preparation method and treatments. Between 3400 and 2800 cm^{-1}, peaks associated with O–H and C–H stretching, characteristic of polysaccharides, are visible. Characteristic bands related to cellulose were also detected in the region between 1650–900 cm^{-1}: a peak at about 1630 cm^{-1} (related to water molecules absorbed in the cellulose); at around 1208 C–OH and C–CH bending, at 1157 cm^{-1} the C–C breathing ring asymmetric stretching, at 1105 cm^{-1} the asymmetric stretching of the C–O–C glycosidic ether band, at 1060 and 1030 cm^{-1} the stretching of the primary and secondary C–OH alcohols, and finally at 897 cm^{-1} the symmetric stretching of the C–O–C often associated to the amorphous region of cellulose [1,2].

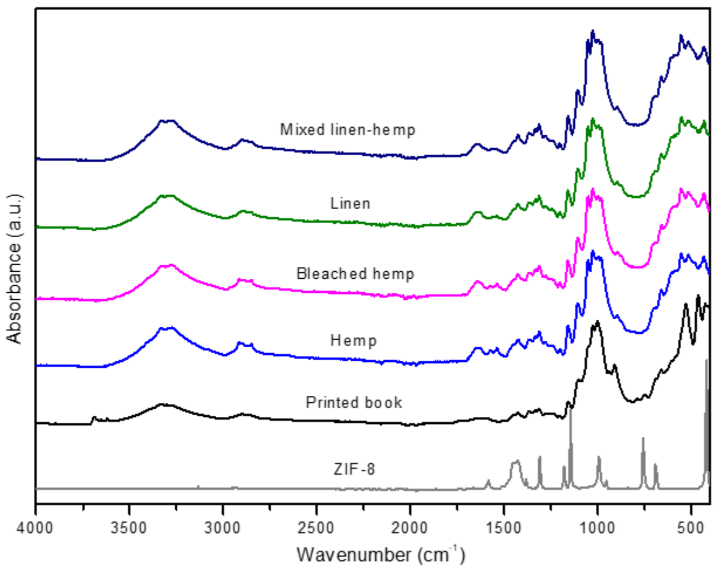

Figure S6. FTIR-ATR spectra of all considered paper and ZIF-8 to identify the main characteristic peaks.

In commercial paper, the use of coating treatments (mixing pigments and additives) is quite common. In the case of the printed book, the two minor peaks present at approximately 3370 and 3610 cm^{-1} can be attributed to clay materials, such as kaolin, which is frequently added to commercial paper to produce a more durable final product [3,4]. The combination of kaolin with adhesive, used to fix the kaolin and create a shiny superficial aspect, could also be hypothesised. The nature of the adhesive, most likely starch derivatives, can be inferred from the literature; however, much of the starch peak is partially covered by the paper signal. The peak at about 1590 cm-1 in the printed book could be associated with the ink used. No differences are evidenced among the bleached and unbleached linen and hemp hand-made papers.

The Zif-8 spectrum is characterised by the C=N bending at 1584 cm-1 from imidazole, the peaks of C-N stretching vibration at 995 cm^{-1}, 1146 cm^{-1}, and 1427 cm^{-1}, and the peak at 421 cm^{-1} representing the Zn-N bonding [5,6].

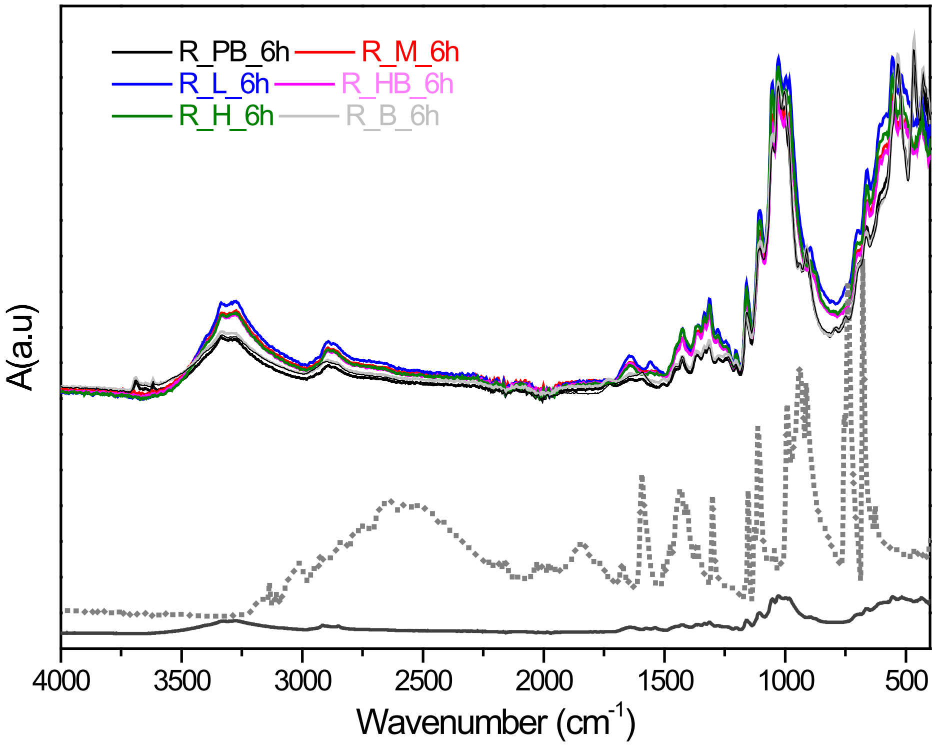

Figure S7. FTIR-ATR spectra obtained for all papers subjected to the the blank experiment (R_h- hemp; R_bh- bleached hemp; R_m – mix hemp/linen; R_l – linen; R_b – book) after 6 hours application time. 2-metyilimidazole and the hemp spectra are reported for comparison.

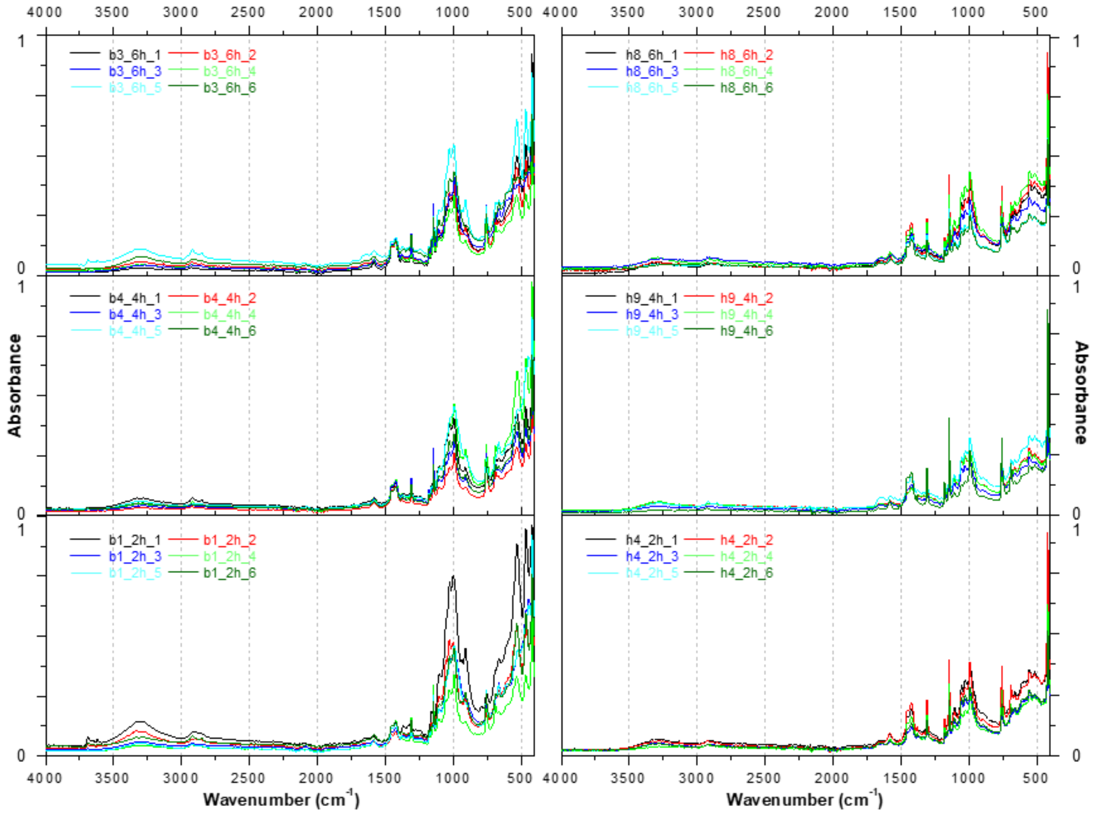

Figure S8. Comparison of FTIR-ATR spectra obtained for multiple points and samples of the printed book and hemp after 2, 4, and 6 hours of treatment with ZIF-8. All spectra in Absorbance mode were normalised to a scale of 0 to 1.

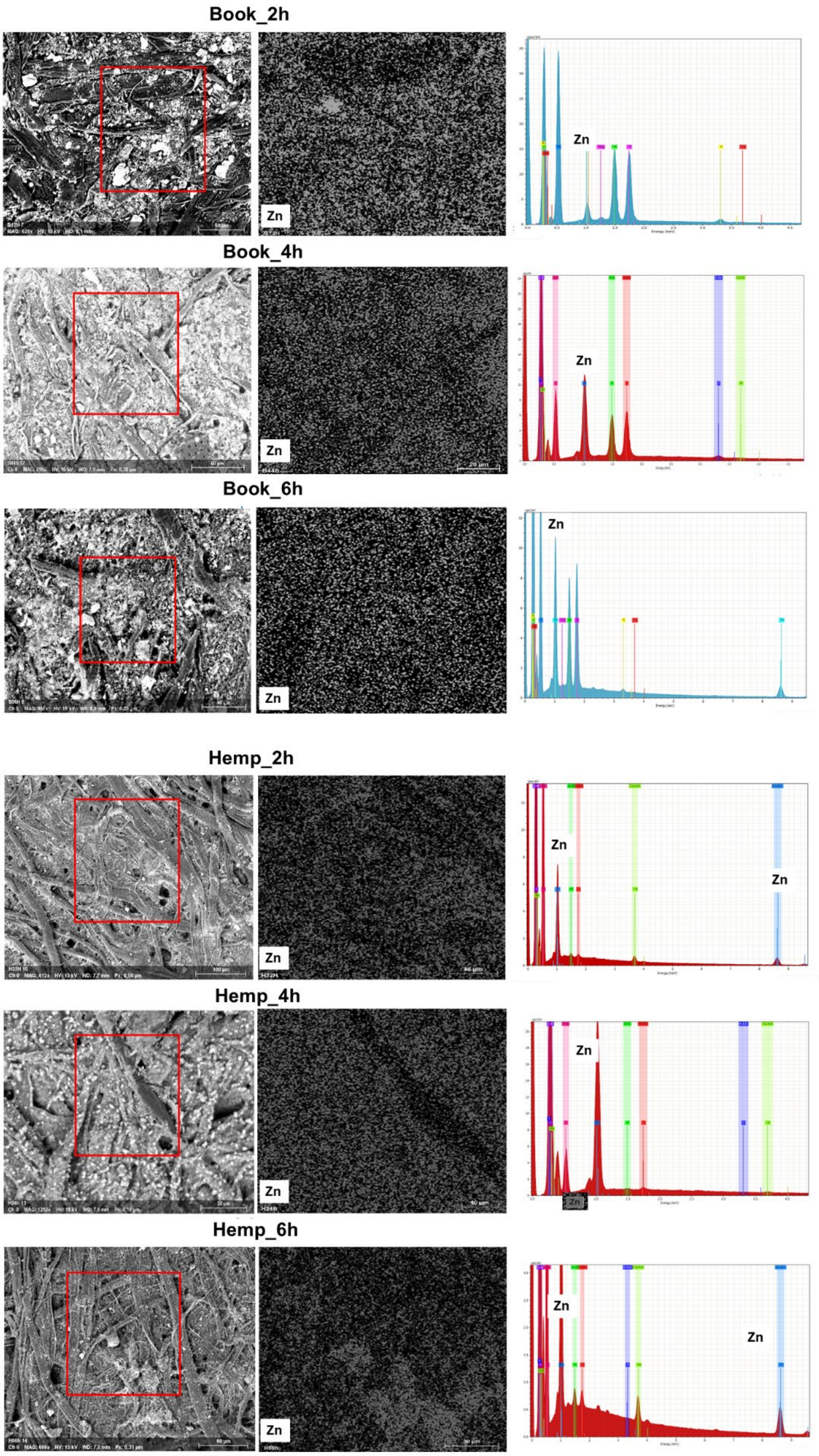

Figure S9. SEM images, Zn distribution maps and EDX spectra on a selected area obtained for hemp and book after 2, 4 and 6h application time.

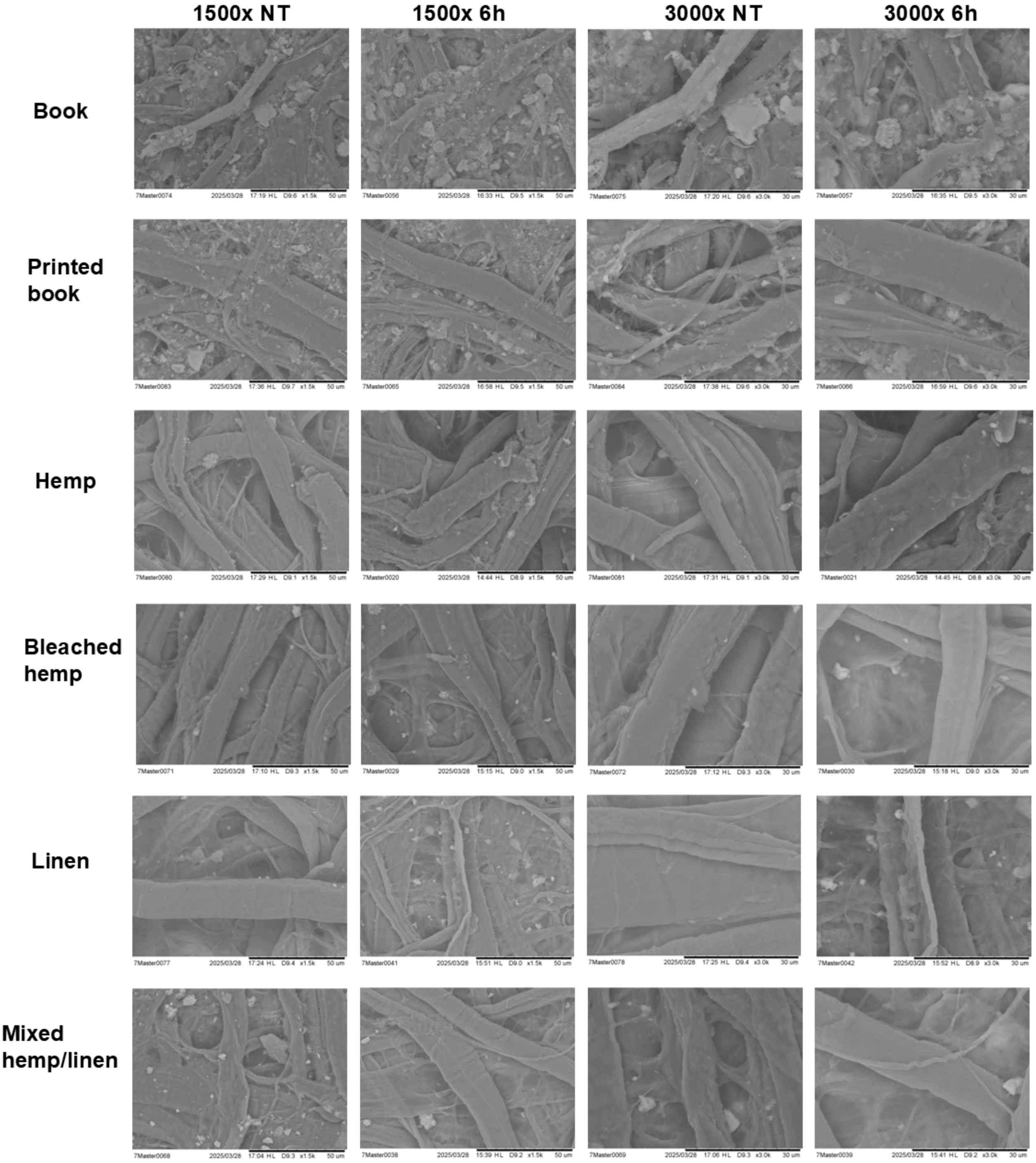

Figure S10. Confront of SEM images (1550x, 3000x) obtained for all papers before (NT) and after 6h contact with the solvent (10 ml solution of 2-HmIM 27.0 mmol).

Table S1. Variations in the chromatic coordinates L*, a*, b*, and ΔE of all paper samples after treatments. All samples were measured on the front and back, considering at least 10 points on three replicates per paper type and application time. The value collected for the papers subjected to the blank experiment are reported as R_name_2/4/6h.

Type of paper	Application time (h)	ΔL	Δa	Δb	ΔE
Hemp	R_h_2h	0.45±0.35	-0.24±0.06	0.45±0.37	0.68±0.46
	R_h_4h	0.35±0.50	-0.32±0.11	0.41±0.47	0.41±0.58
	R_h_6h	0.41±0.47	-0.30±0.10	0.20±0.56	0.20±0.47
	h_2h	0.71±0.18	0.19±0.01	0.21±0.22	0.80±0.30
	h_4h	0.17±0.04	0.07±0.04	0.33±0.17	0.72±0.26
	h_6h	0.33±0.10	0.04±0.01	0.15±0.01	0.43±0.15
Bleached hemp	R_bh_2h	-0.25±0.36	-0.02±0.04	-0.73±0.79	0.94±0.63
	R_bh_4h	-0.09±0.13	-0.10±0.03	0.02±0.20	0.24±0.12
	R_bh_6h	-0.31±0.32	-0.11±0.02	-0.22±0.38	0.56±0.29
	bh_2h	0.55±0.00	0.36±0.00	-0.82±0.17	1.08±0.33
	bh_4h	0.76±0.10	0.31±0.04	-1.29±0.05	1.53±0.25
	bh_6h	0.53±0.06	0.28±0.00	-0.61±0.05	0.86±0.21
Mix hemp/linen	R_m_2h	1.21±0.57	-0.44±0.13	-1.34±0.32	1.89±0.58
	R_m_4h	0.63±0.70	-0.36±0.13	-0.73±0.54	1.17±0.69
	R_m_6h	0.77±0.89	-0.33±0.16	-0.61±0.61	1.30±0.75
	m_2h	0.63±0.07	0.07±0.05	-0.19±0.05	0.69±0.18
	m_4h	0.63±0.20	0.10±0.01	-0.43±0.05	0.80±0.29
	m_6h	0.35±0.01	0.11±0.01	0.35±0.11	0.68±0.32
Linen	R_l_2h	-0.27±0.15	0.12±0.03	-1.21±0.66	1.24±0.66
	R_l_4h	0.04±0.15	-0.02±0.05	-0.84±0.08	0.85±0.05
	R_l_6h	0.21±0.15	0.01±0.05	-1.02±0.63	1.04±0.61
	l_2h	0.41±0.02	0.39±0.01	-0.15±0.17	0.61±0.30
	l_4h	0.08±0.12	0.28±0.02	0.05±0.03	0.40±0.18
	l_6h	0.52±0.07	0.25±0.02	0.14±0.11	0.70±0.16
Book	R_b_2h	2.33±0.99	1.21±0.51	0.72±0.87	2.72±1.18
	R_b_4h	2.75±0.38	1.59±0.14	0.21±0.36	3.18±0.44
	R_b_6h	3.14±0.29	0.72±0.06	0.42±0.38	3.25±0.42
	b_2h	2.66±0.09	0.33±0.03	0.01±0.17	2.72±0.36
	b_4h	3.02±0.01	0.40±0.01	-0.25±0.34	3.24±0.52
	b_6h	3.29±0.23	0.45±0.05	0.52±0.19	3.47±0.51

References

1. Garside, P.; Wyeth, P. Identification of Cellulosic Fibres by FTIR Spectroscopy - Thread and Single Fibre Analysis by Attenuated Total Reflectance. *Stud. Conserv.* **2003**, *48*, 269–275, doi:10.1179/SIC.2003.48.4.269.
2. Paro, E.; Benvestito, C.; Pugliese, S.; Izzo, F.C.; Balliana, E.; Zendri, E. Study and

characterization of paper bookbindings from 16 to 18th stored in the Marciana National Library (Venice). *Herit. Sci.* **2024**, *12*, 1–14, doi:10.1186/s40494-024-01339-6.

3. Conners, T.E.; Banerjee, S. *Surface Analysis of Paper*; 1st ed.; CRC Press, 2020; ISBN 9780429279997.

4. Canals, T.; Riba, J.R.; Cantero, R.; Cansino, J.; Domingo, D.; Iturriaga, H. Characterization of paper finishes by use of infrared spectroscopy in combination with canonical variate analysis. *Talanta* **2008**, *77*, 751–757, doi:10.1016/J.TALANTA.2008.07.059.

5. Ran, J.; Chen, H.; Bi, S.; Guo, Q.; Deng, Z.; Cai, G.; Cheng, D.; Tang, X.; Wang, X. One-step in-situ growth of zeolitic imidazole frameworks-8 on cotton fabrics for photocatalysis and antimicrobial activity. *Cellulose* **2020**, *27*, 10447–10459, doi:10.1007/S10570-020-03483-1/FIGURES/9.

6. Di Matteo, V.; Di Filippo, M.F.; Ballarin, B.; Gentilomi, G.A.; Bonvicini, F.; Panzavolta, S.; Cassani, M.C. Cellulose/Zeolitic Imidazolate Framework (ZIF-8) Composites with Antibacterial Properties for the Management of Wound Infections. *J. Funct. Biomater.* **2023**, *14*, 472–487, doi:10.3390/jfb14090472.
